# Mode of delivery and postpartum HIV-1 disease progression and mortality in a Kenyan cohort

**DOI:** 10.1186/1471-2393-14-257

**Published:** 2014-08-03

**Authors:** Jennifer A Unger, Barbra A Richardson, Phelgona A Otieno, Carey Farquhar, Dalton Wamalwa, Grace C John-Stewart

**Affiliations:** Department of Obstetrics and Gynecology, University of Washington, Seattle, Washington USA; Department of Biostatistics, University of Washington, Seattle, Washington USA; Department of Global Health, University of Washington, Seattle, Washington USA; Centre for Clinical Research, Kenya Medical Research Institute, Nairobi, Kenya; Department of Medicine, University of Washington, Seattle, Washington USA; Department of Epidemiology, University of Washington, Seattle, Washington USA; Department of Pediatrics, University of Nairobi, Nairobi, Kenya; Department of Pediatrics, University of Washington, Seattle, Washington USA; Department of Obstetrics and Gynecology, University of Washington, Harborview Medical Center, Box 359865, 325 Ninth Ave, Seattle, Washington 98195 USA

**Keywords:** HIV, Mode of delivery, Cesarean section, HIV-1 disease progression, Maternal mortality

## Abstract

**Background:**

There are limited data on the impact of cesarean section delivery on HIV-1 infected women in Sub-Saharan Africa. The purpose of this study was to assess the effect of mode of delivery on HIV-1 disease progression and postpartum mortality in a Kenyan cohort.

**Methods:**

A prospective cohort study was conducted in Nairobi, Kenya from 2000–2005. We determined changes in CD4^+^ counts, HIV-1 RNA levels and mortality during the first year postpartum between HIV-1 infected women who underwent vaginal delivery (VD), non-scheduled cesarean section (NSCS) and scheduled cesarean section (SCS) and received short-course zidovudine. Loess curves and multivariate linear mixed effects models were used to compare longitudinal changes in maternal HIV-1 RNA and CD4^+^ counts by mode of delivery. Kaplan Meier curves, the log rank test, and Cox proportional hazards regression were used to assess difference in mortality.

**Results:**

Of 501 women, 405 delivered by VD, 74 delivered by NSCS and 22 by SCS. Baseline characteristics were similar between the VD and NSCS groups. Baseline antenatal CD4^+^ counts were lowest and HIV-1 RNA levels highest in the NSCS group but HIV-1 RNA levels were similar between groups at delivery. The rate of decline in CD4^+^ cells and rate of increase in HIV-1 RNA did not differ between groups. After adjusting for confounders, women who underwent NSCS had a 3.39-fold (95% CI 1.11, 10.35, P = 0.03) higher risk of mortality in the first year postpartum compared to women with VD.

**Conclusions:**

Non-scheduled cesarean section was an independent risk factor for postpartum mortality in HIV-1 positive Kenyan women. The cause of death was predominantly due to HIV-1 related infections, and not direct maternal deaths, however, this was not mirrored by differential changes in HIV-1 progression markers between the groups.

## Background

Cesarean section prior to the onset of labor remains an effective intervention for the prevention of mother-to-child transmission (PMTCT) of HIV-1 among HIV-1 infected women receiving either no antiretrovirals (ARVs) or short-course ARVs with incompletely suppressed viral levels [1]. However, surgical delivery may have detrimental effects, particularly among untreated or incompletely treated HIV-1 infected women in sub-Saharan Africa (SSA). In 2012 WHO, UNAIDS and UNICEF estimated that only 62% of the 1.4 to 1.7 million pregnant women living with HIV were receiving the most effective antiretroviral PMTCT regimen [2].

Women with HIV-1 have higher rates of postpartum morbidity compared to uninfected women regardless of mode of delivery but morbidity is significantly increased with a cesarean delivery [3–5]. Moreover, postpartum morbidity appears to be highest in HIV-1 infected women undergoing an emergent or non-scheduled cesarean delivery [6]. One study from Latin America and Caribbean found that postpartum morbidity was approximately 3-fold higher among women having non-scheduled cesarean section (NSCS) than that those with vaginal deliveries (VD). In the same study, morbidity among women with scheduled cesarean section (SCS) was similar to that of vaginal deliveries [7]. The etiology of morbidity following cesarean delivery is multifactorial, and includes severe anemia [3, 8], fever [5, 6, 8], pneumonia [3], UTI [8], and endometritis [5, 8, 9].

There are conflicting data regarding the effect of mode of delivery on HIV-1 disease progression and mortality. Published studies examining the relationship between mode of delivery and disease progression have, thus far, been conducted in North American and European cohorts. The WITS study, conducted in a North American cohort, found no relationship between mode of delivery and subsequent HIV-1 disease progression [10]. In contrast, the European Collaborative Study found less subsequent symptomatic HIV-1 disease among women who underwent a vaginal delivery compared to scheduled cesarean section [11].

To date no study has evaluated mode of delivery as a cofactor for HIV-1 disease progression in sub-Saharan Africa, where rates of maternal morbidity and mortality are higher in general and treatment guidelines and access differ. The purpose of this study was to determine the effect of mode of delivery on HIV-1 disease progression as defined by decreases in CD4^+^ counts and increases in HIV-1 viral load and the effect on mortality in HIV-1 infected women during the first year postpartum.

## Methods

### Study setting and design

This study utilized data from a historical cohort study conducted in Nairobi, Kenya from October 2000 to June 2005. Women were recruited from 4 Nairobi City Council clinics when they presented for antenatal care prior to 28 weeks gestation. HIV-1 seropositive women were eligible to participate in the study if they planned to reside in Nairobi for 2 years after the birth of their infant. Written informed consent was obtained from all participants. Ethical approval was obtained from the Institutional Review Board of the University of Washington and Ethical Review Committee at the Kenya Medical Research Institute.

The purpose of this sub-analysis was to determine the effect of mode of delivery on HIV-1 maternal disease progression as defined by decreases in CD4^+^ counts, increases in HIV-1 RNA levels and risk of death in the first year postpartum. The main aim of the larger study was to determine whether the presence of HIV-specific cytotoxic T lymphocyte (CTL) responses in children born to HIV-1 positive women were associated with subsequent protection from infection [12].

Pregnant participants received routine antenatal care and a short-course zidovudine regimen, consisting of 300 mg orally twice a day from 36 weeks’ gestation until onset of labor and 300 mg every 3 hours from onset of labor until delivery, to reduce maternal to child transmission of HIV-1. This protocol was standard of care at the time of study [13]. Clinical, immunologic and virologic characteristics were assessed at 32 weeks gestation as baseline. At delivery, blood was collected to measure HIV-1 RNA levels. Women were assessed at postpartum months 1, 3, 6, 9, and 12 for BMI, CD4^+^ T lymphocyte counts and HIV-1 RNA levels. Women received both multivitamins and iron for the first 6 months postpartum. Those with CD4^+^ cell counts <200 cells/uL received cotrimoxazole prophylaxis. When highly active antiretroviral therapy (ART) became more widely available in Kenya in 2003, women were referred to treatment programs and started on therapy.

Women delivered at Kenyatta National Hospital with a certified nurse midwife or obstetrician. Decisions for mode of delivery were made at the discretion of KNH medical personnel and, during the study period, were not based on HIV-RNA levels. Cesarean sections performed prior to the onset of labor or rupture of membranes were defined as scheduled cesarean sections (SCS). A non-scheduled cesarean section (NSCS) was defined as one performed after the onset of labor or rupture of membranes.

### CD4^+^ and HIV RNA analyses

CD4^+^ cell counts were performed at the University of Nairobi using the FACScan flow cytometer (Becton Dickinson, Mountain View, California, USA). Plasma HIV-1 RNA levels were quantified by the use of transcription mediated amplification assay (Gen-Probe) at the Fred Hutchinson Cancer Research Center [14].

### Statistical analysis

Maternal baseline characteristics were compared by mode of delivery using the Student’s t-test for continuous variables and Pearson’s chi-square tests for categorical variables. The vaginal delivery (VD) group was the reference group for all comparisons. To describe and compare longitudinal changes in maternal HIV-1 RNA levels and CD4^+^ lymphocyte counts we used Loess curves and multivariate linear mixed effects models controlling for baseline levels of age, antenatal weight, partnership, antenatal CD4^+^, antenatal HIV-1 RNA, BMI and employment. Kaplan Meier curves, the log rank test, and Cox proportional hazards regression were used to determine if mode of delivery was associated with time to death during the first year postpartum. In all study analyses, P ≤ 0.05 was considered significant. All data were analyzed using STATA Software (StataCorp. 2011. *Stata Statistical Software: Release 12*. College Station, TX: StataCorp LP).

## Results

### Characteristics of participants according to mode of delivery

Delivery information was available for 501 (94%) of the 535 participants in the cohort. Of these, 405 (81%) delivered vaginally and 22 (4%) by SCS (Table 1). Seventy-four women (15%) delivered by NSCS. The percentage of women delivered by SCS increased over the study duration, the majority occurring in the last two years when practice changes regarding SCS for PMTCT started in Kenya (Table 2). This, however, was not standard practice among all practitioners.

**Table 1:** **Characteristics of the study population by mode of delivery (n = 501 deliveries)**

Charateristics	Vaginal delivery (n = 405)	Non-scheduled cesarean section (n = 74)	P (NSCS vs. VD) ^a^	Scheduled cesarean section (n = 22)	P (SCS vs. VD) ^a^
	Mean (95% CI) or % (n)	Mean (95% CI) or % (n)		Mean (95% CI) or % (n)	
Age at enrollment (yr)	25.1 (24.6, 25.5)	25.8 (24.8, 26.9)	0.2	27.8 (25.7, 29.9)	0.006
Prenatal weight (kg)	63.5 (62.6, 64.4)	62.9 (61.0, 62.7)	0.6	70.4 (65.1, 75.7)	0.001
Prior Pregnancies	1.5 (1.4, 1.6)	1.4 (1.1, 1.7)	0.5	1.9 (1.1, 2.7)	0.1
Gestational Age (wk)	39.2 (39.0, 39.4)	39.7 (39.3, 40.2)	0.03	38.4 (37.1, 39.7)	0.2
Years of Education	8.9 (8.6, 9.2)	8.9 (8.3, 9.6)	0.9	9.6 (8.7, 10.5)	0.2
Lifetime Sexual Partners	3.1 (2.9, 3.3)	5.0 (2.0, 7.9)	0.2	3.4 (2.4, 4.4)	0.5
Years in Relationship	4.8 (4.4, 5.2)	5.7 (4.5, 7.0)	0.7	6.8 (3.9, 9.6)	0.2
Stable partnership	97% (382/394)	85% (62/73)	<0.001	86% (19/22)	0.04
Employed	29% (113/394)	37% (27/73)	0.2	55% (12/22)	0.01
Length of Labor (hrs)	9.8 (9.2, 10.3)	11.9 (9.9, 13.8)	0.04	N/A	
Duration of Rupture of Membranes (hrs)	4.5 (3.2, 5.9)	6.5 (3.9, 9.0)	0.2	N/A	
Doses of zidovudine	2.8 (2.6, 3.0)	3.7 (3.1, 4.3)	0.004	2.5 (1.8, 3.2)	0.5
Antenatal BMI	24.5 (24.2, 24.8)	25.0 (24.4, 25.6)	0.2	27.0 (25.1, 28.8)	<0.001
Antenatal Hgb	10.5 (10.3, 10.7)	10.5 (10.2, 10.8)	1.0	10.9 (10.5, 11.3)	0.2
Antenatal CD4 count	486.6 (460.4, 512.7)	418.9 (363.4, 474.4)	0.04	456.1 (371.5, 540.7)	0.6
Antenatal HIV-1 RNA log_10_ copies/ml	4.6 (4.5, 4.7)	4.8 (4.6, 5.0)	0.05	4.7 (4.4, 5.0)	0.7
Delivery HIV-1 RNA log_10_ copies/ml	4.0 (3.9, 4.1)	4.1 (3.9, 4.4)	0.5	3.6 (3.2, 4.1)	0.06

^a^P value from a Student’s t-test for continuous variables and Pearson’s chi-square tests for categorical variables (statistical significance, α = 0.05).

**Table 2:** **Mode of delivery by study year**

Year	VD	NSCS	SCS	Total
2000	86.4% (38)	13.6% (6)	0% (0)	44
2001	89.7% (130)	7.6% (11)	2.8% (4)	145
2002	79.3% (130)	17.6% (29)	3.0% (5)	164
2003	71.4% (90)	20.6% (26)	7.9% (10)	126
2004	76.2% (16)	4.8% (1)	19% (4)	21

VD = vaginal delivery.

NSCS = non-scheduled cesarean section.

SCS = scheduled cesarean section.

The women who underwent vaginal deliveries and NSCS were similar in age, BMI, years of education, employment and number of prior pregnancies. However, those who delivered via NSCS were less likely to be in a stable relationship (P < 0.001). Women who delivered by SCS were generally older (P = 0.006), had higher BMI (P < 0.001) and were more likely to be employed (P = 0.01) than those who delivered vaginally.

Women undergoing NSCS had significantly longer labors than those with a vaginal delivery (P = 0.04). However duration of rupture of membranes was similar between the two groups. The mean enrollment CD4^+^ cell count of the cohort (~32 weeks gestation) was 451 cells/mm^3^ and lower among the NSCS group compared to the vaginal delivery group (-67.7, P = 0.04). Mean HIV-1 RNA copy number was also significantly higher in those who underwent NSCS compared to the vaginal delivery group (4.8 versus 4.6 log_10_ copies per ml, P = 0.05).

HIV-1 RNA levels were lower for all three groups at delivery compared to earlier in pregnancy, due to short-course zidovudine. Overall women received an average of 3 doses of zidovudine during labor but the greatest number  was received by the NSCS group. HIV-1 RNA levels did not differ between groups at delivery.

### Indication for cesarean section

The most common indication for NSCS was obstructed labor (47%). The data regarding indication for cesarean section were not complete for either the SCS or NSCS groups but this was particularly true for majority of SCS (68%) as indicated in Table 3.

**Table 3:** **Indications for cesarean section in NSCS and SCS groups (n = 96)**

	Non-scheduled cesarean section (n = 74)% (n)	Scheduled cesarean section (n = 22)% (n)
Obstructed labor	47 (35)	(0)
Fetal distress	14 (10)	9 (2)
Malpresentation	5 (4)	5 (1)
Abruption	4 (3)	9 (2)
Elective	(0)	9 (2)
Unknown	30 (22)	68 (15)

### Postpartum immunologic progression

**Figure 1:**
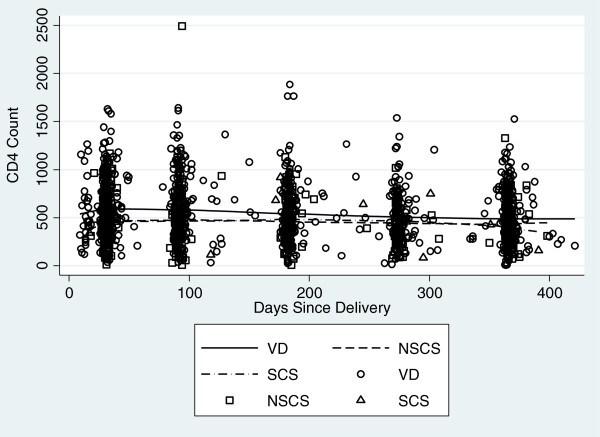
**CD4** ^**+**^ **lymphocyte count over first year postpartum by mode of delivery.**

Postpartum CD4^+^ counts were available for 450 women; 365 (92%) of VD group, 74 (100%) of NSCS group and 17 (77%) of SCS group. The mean change per month for all women was -8.70 cells/ul (95% CI: -10.53, -6.87, P < 0.001). At the first month postpartum visit, women who delivered via NSCS had mean CD4^+^ counts 97.90 cell/ul lower than those with a VD (95% CI: -172.22, -23.57, P = 0.01) and women with a SCS at month one post partum had mean CD4^+^ counts 112.88-cells/ul lower than those with a VD (95% CI: -197.14, -28.61, P = 0.009). There was not a significant difference in change in CD4^+^ count over time for the NSCS group compared to the vaginal delivery group or for those with a SCS compared to a vaginal delivery. (Figure 1) CD4^+^ count comparisons between groups remained similar after controlling for potential confounding factors of age, antenatal weight, stable partnership, antenatal CD4^+^ count, antenatal HIV-1 RNA, antenatal BMI, and employment.

### Postpartum virologic progression

**Figure 2:**
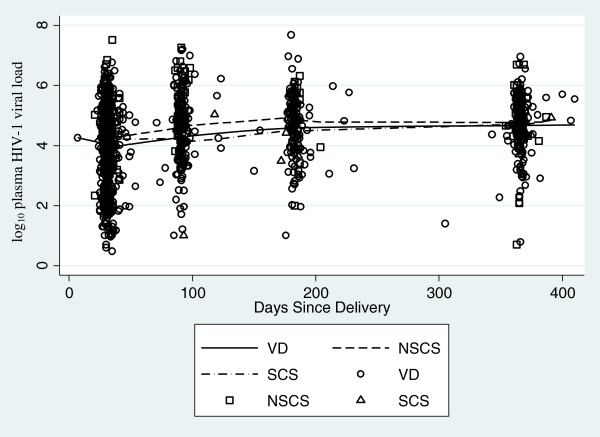
**HIV RNA copy number (log**_**10**_ **) over first year postpartum by mode of delivery.**

HIV-1 RNA measures were available for 475 women during the first year postpartum; 383 (95%) of VD group, 70 (95%) of NSCS group and 22 (100%) of SCS group. At one month postpartum women who delivered by NSCS had a mean viral load that was 0.34 log_10_ copies/ul higher than women with a VD (95% CI: 0.05, 0.63, P = 0.02). In contrast, women in the SCS group had a similar postpartum viral load as the VD group. There was not a significant difference in viral load over time for women having a NSCS compared to VD or for those with a SCS versus VD (Figure 2). The mean change per month over the first year for all women was 0.06 log_10_ copies/ul (95% CI: 0.05, 0.07, P < 0.001). Results regarding viral load change over time were similar after controlling for potential confounding factors.

### Postpartum mortality

**Figure 3:**
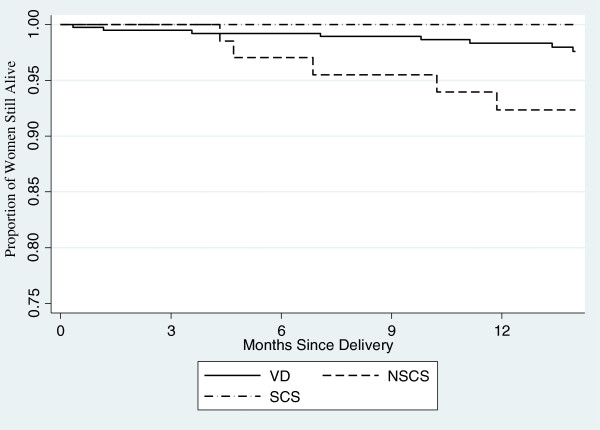
**Proportion of women still alive over first year postpartum by mode of delivery.**

There were a total of 13 deaths in the first year postpartum, 8 in the VD group, 5 in the NSCS group, 0 in the SCS group. The 12-month post-delivery mortality risk was significantly higher for women having a NSCS compared to those with VD (P = 0.02) (Figure 3). Women in the NSCS group had a 3.39-fold increased risk of mortality versus VD (95% CI 1.11, 10.35, P = 0.03). Results were similar when controlling for potential confounding factors including age, antenatal weight, stable partnership, employment, length of labor, duration of rupture of membranes, baseline CD4^+^ count, baseline HIV-1 RNA, and baseline BMI.

Causes of death in all groups were most often AIDS related (n = 11), including meningitis, bacterial pneumonia and tuberculosis (TB) (Table 4). There was one death from a pelvic abscess in the vaginal delivery group, which occurred 9 months after delivery. In the NSCS group, 3 of the 5 women who died did so from confirmed AIDS related illnesses. The other two deaths were from unknown causes though one woman had a CD4^+^ count less than 100 at the visit prior to her death and the other woman was reported to have Kaposi’s sarcoma at the time of her death.

**Table 4:** **Delivery type and cause of death in cases of first year postpartum mortality**

Mortality case	Delivery type	Indication for NSCS	Year of death	Months post partum	Last CD4 count	Cause of death
1	VD	n/a	2000	1	125	Pneumonia
2	VD	n/a	2000	9	1011	Sepsis/Abscess
3	VD	n/a	2001	7	204	Tuberculosis
4	VD	n/a	2002	12	141	Unknown
5	VD	n/a	2003	11	187	Meningitis
6	VD	n/a	2001	12	106	Pneumonia
7	VD	n/a	2002	10	65	Tuberculosis
8	VD	n/a	2003	4	176	Pneumonia
9	NSCS	Unknown	2000	5	56	Tuberculosis
10	NSCS	Fetal distress	2002	11	5	Pneumonia
11	NSCS	Arrest of labor	2002	7	*	Unknown**
12	NSCS	Arrest of labor	2002	10	76	Unknown
13	NSCS	Unknown	2003	4	275	Meningitis

VD = vaginal delivery, NSCS = non-scheduled cesarean section.

*No last CD4^+^ reported.

**Reported to have Kaposi’s sarcoma at time of death.

## Discussion and conclusions

In this study of HIV-1 infected women in Kenya, we found that mode of delivery was associated with mortality in the first year postpartum. The greatest risk of death was among women who underwent a non-scheduled cesarean section (NSCS) and no deaths occurred in the group of women who underwent a scheduled cesarean section (SCS). Baseline and postpartum differences in CD4^+^ lymphocyte count and HIV-1 RNA levels did not explain the 3-fold greater risk of mortality observed in the NSCS group. Although mortality was increased among women with NSCS, there were no significant differences in CD4^+^ cell count decline or HIV-1 RNA levels between the three mode of delivery groups over the 1-year postpartum period. Thus, although mode of delivery appeared to have an effect on mortality, the mortality difference between groups was not mirrored by similar effects of delivery mode on traditional markers of HIV-1 disease progression. Specifically, neither NSCS nor SCS were associated with a significant decrease in CD4^+^ counts or increase in HIV-1 RNA levels.

The causes of death among the women were related to infections and not obstetrical causes, typically occurring after 3 months postpartum. The women who died did have advanced HIV-1 as measured by CD4^+^ <200, although this was true among deaths in all groups. The average maternal mortality rate (MMR) at Kenyatta National Hospital at the time of the study was approximately 300 per 100,000 live births [15] similar to the countrywide rate of 488 per 100,000 live births in 2008c [16]. The most common causes of maternal deaths in Kenya continue to be maternal hemorrhage and infection, with HIV-1 being a significant indirect contributor.

Previous studies have demonstrated conflicting results when examining the effects of mode of delivery on disease progression among HIV-1 infected women in US and European studies. The European Collaborative Study (ECS) of over 1200 women found more symptomatic HIV-1 among women who delivered by cesarean section than those who delivered vaginally [11]. However, the Women and Infants Transmission Study (WITS) did not find a significant association between mode of delivery and disease progression, defined by a decrease in CD4% and HIV-1 RNA increase during the 18 months post delivery, or with clinical progression or death [10]. These studies examined similar virologic, immunologic and mortality outcomes as our study. Although the WITS trial took place during a similar time period (1990–2004) as our study (2000–2005), the majority of women received HAART after 1996, which would be expected to attenuate impact of mode of delivery on HIV-1 progression.

Currently in sub-Saharan Africa the estimated coverage of pregnant women living with HIV-1, receiving the most effective antiretroviral (ARV) regimens for prevention of mother to child transmission is 62% while approximately 10% continue to receive only single-dose nevirapine [2]. Although advanced ARVs may greatly influence maternal outcomes, the lack of coverage continues to suggest that HIV-1 infected pregnant women will still be affected by their mode of delivery and that long labors followed by surgery may be deleterious for this population. When caring for HIV-1 positive women in labor, the risks of cesarean delivery after prolonged labor must be considered when making decisions about labor management.

Other types of major surgery in HIV-1 infected individuals have been associated with higher rates of mortality than in HIV non-infected patients, even when infected individuals are on highly active antiretroviral therapy (HAART) [17]. Chronic immune activation has been associated with HIV disease progression independent of cellular markers and previous studies have demonstrated that trauma and surgery stimulate immune activation [18–20]. Major abdominal surgery following labor likely increases immune activation to a greater extent than a vaginal delivery or planned cesarean section alone and thus immune activation may be a contributor to mortality in this cohort. Although this cannot be concluded from the available data it is worthy of exploration in future studies.

There are several strengths and limitations to this study. The study was primarily designed to examine maternal HIV-1 disease progression, and included longitudinally collected immunologic and virologic markers of disease progression including CD4^+^ and HIV-1 RNA levels. Limitations of this study include the non-randomized design; we noted several differences at baseline between the three delivery groups and although these were included in multivariable models, it is possible that there was residual confounding. The study was also not originally designed to specifically examine mode of delivery as a factor in disease progression and we had incomplete delivery data. The number of women living with HIV-1 who are receiving HAART is currently higher than at the time of this cohort. HAART improves maternal outcomes and may influence the effect that mode of delivery plays on HIV-1 disease. Data regarding postpartum morbidity was also limited and made it difficult to assess its role in this cohort.

In summary we found that NSCS appears to be a risk factor for HIV-1 infected women during the first year postpartum. These findings may influence postpartum surveillance in the non-scheduled cesarean section population but given the limitations of the data it is not possible to make recommendations regarding mode of delivery or a change in labor management. Future studies should include mode of delivery as a factor in models examining maternal deaths attributable to HIV and explore a design to prospectively examine HIV-1 infected women who undergo non-scheduled cesarean section.

## References

[1] Read JS, Newell MK (2005). Efficacy and safety of cesarean delivery for prevention of mother-to-child transmission of HIV-1. Cochrane Database Syst Rev.

[2] World Health Organization., UNAIDS., UNICEF (2011). Global HIV/AIDS Response: Epidemic Update and Health Sector Progress Towards Universal Access: Progress Report 2011.

[3] Ferrero S, Bentivoglio G (2003). Post-operative complications after caesarean section in HIV-infected women. Arch Gynecol Obstet.

[4] Lapaire O, Irion O, Koch-Holch A, Holzgreve W, Rudin C, Hoesli I (2006). Increased peri- and post-elective cesarean section morbidity in women infected with human immunodeficiency virus-1: a case-controlled multicenter study. Arch Gynecol Obstet.

[5] Fiore S, Newell ML, Thorne C (2004). Higher rates of post-partum complications in HIV-infected than in uninfected women irrespective of mode of delivery. AIDS.

[6] Marcollet A, Goffinet F, Firtion G, Pannier E, Le Bret T, Brival ML, Mandelbrot L (2002). Differences in postpartum morbidity in women who are infected with the human immunodeficiency virus after elective cesarean delivery, emergency cesarean delivery, or vaginal delivery. Am J Obstet Gynecol.

[7] Duarte G, Read JS, Gonin R, Freimanis L, Ivalo S, Melo VH, Marcolin A, Mayoral C, Ceriotto M, de Souza R, Cardoso E, Harris DR, NISDI Perinatal Study Group (2006). Mode of delivery and postpartum morbidity in Latin American and Caribbean countries among women who are infected with human immunodeficiency virus-1: the NICHD International Site Development Initiative (NISDI) Perinatal Study. Am J Obstet Gynecol.

[8] Read JS, Tuomala R, Kpamegan E, Zorrilla C, Landesman S, Brown G, Vajaranant M, Hammill H, Thompson B (2001). Mode of delivery and postpartum morbidity among HIV-infected women: the women and infants transmission study. J Acquir Immune Defic Syndr.

[9] Urbani G, de Vries MM, Cronje HS, Niemand I, Bam RH, Beyer E (2001). Complications associated with cesarean section in HIV-infected patients. Int J Gynaecol Obstet.

[10] Navas-Nacher EL, Read JS, Leighty RM, Tuomala RE, Zorrilla CD, Landesman S, Rosenblatt H, Hershow RC (2006). Mode of delivery and postpartum HIV-1 disease progression: the Women and Infants Transmission Study. AIDS.

[11] The European Collaborative Study (1994). Caesarean section and risk of vertical transmission of HIV-1 infection. The European Collaborative Study. Lancet.

[12] John-Stewart GC, Mbori-Ngacha D, Payne BL, Farquhar C, Richardson BA, Emery S, Otieno P, Obimbo E, Dong T, Slyker J, Nduati R, Overbaugh J, Rowland-Jones S (2009). HV-1-specific cytotoxic T lymphocytes and breast milk HIV-1 transmission. J Infect Dis.

[13] Shaffer N, Chuachoowong R, Mock PA, Bhadrakom C, Siriwasin W, Young NL, Chotpitayasunondh T, Chearskul S, Roongpisuthipong A, Chinayon P, Karon J, Mastro TD, Simonds RJ (1999). Short-course zidovudine for perinatal HIV-1 transmission in Bangkok, Thailand: a randomised controlled trial. Bangkok Collaborative Perinatal HIV Transmission Study Group. Lancet.

[14] Emery S, Bodrug S, Richardson BA, Giachetti C, Bott MA, Panteleeff D, Jagodzinski LL, Michael NL, Nduati R, Bwayo J, Kreiss JK, Overbaugh J (2000). Evaluation of performance of the Gen-Probe human immunodeficiency virus type 1 viral load assay using primary subtype A, C, and D isolates from Kenya. J Clin Microbiol.

[15] Oyieke JB, Obore S, Kigondu CS (2006). Millennium development goal 5: a review of maternal mortality at the Kenyatta National Hospital, Nairobi. East Afr Med J.

[16] National Council for Population and Development (Kenya), Kenya. Central Bureau of Statistics., Macro International (1999). Kenya Demographic and Health Survey, 1998.

[17] Horberg MA, Hurley LB, Klein DB, Follansbee SE, Quesenberry C, Flamm JA, Green GM, Luu T (2006). Surgical outcomes in human immunodeficiency virus-infected patients in the era of highly active antiretroviral therapy. Arch Surg.

[18] Hazenberg MD, Otto SA, van Benthem BH, Roos MT, Coutinho RA, Lange JM, Hamann D, Prins M, Miedema F (2003). Persistent immune activation in HIV-1 infection is associated with progression to AIDS. AIDS.

[19] Marik PE, Flemmer M (2012). The immune response to surgery and trauma: Implications for treatment. J Trauma Acute Care Surg.

[20] Fahey JL, Taylor JM, Detels R, Hofmann B, Melmed R, Nishanian P, Giorgi JV (1990). The prognostic value of cellular and serologic markers in infection with human immunodeficiency virus type 1. N Engl J Med.

[CR21] The pre-publication history for this paper can be accessed here:http://www.biomedcentral.com/1471-2393/14/257/prepub

